# Supplementary material for: Heat adaptation of phage T7 under an extended genetic code
Source: Virus Evol. 2021 Dec 1;7(2):veab100. doi: 10.1093/ve/veab100 (PMC8923235; doi:10.1093/ve/veab100)
Supplement: veab100_Supp [file veab100_supp.zip › Sequencing_Analysis.zip!/Sequencing_Analysis/A2/evidence/RA_16.html]

BRESEQ :: Evidence


| Read alignment evidence... | | | | | | | | | | | |
| --- | --- | --- | --- | --- | --- | --- | --- | --- | --- | --- | --- |
|  | seq id | position | | ref | new | freq | score (cons/poly) | reads | annotation | genes | product |
| \* | NC\_001604.1 | 3,371 | 0 | C | T | 31.8% | 479.6 / 213.0 | 380 | intergenic (–/–) | *–/–* | –/– |
| Reads supporting (aligned to +/- strand):  **ref** base C (114/145);  **new** base T (59/62);  **total** (173/207) | | | | | | | | | | | |
| Fisher's exact test for biased strand distribution *p*-value = 4.39e-01 | | | | | | | | | | | |
| Kolmogorov-Smirnov test that lower quality scores support variant *p*-value = 5.16e-01 | | | | | | | | | | | |

Only 100 of 382 total aligned reads displayed.  

`GCAACTTGACGCAATGTTAATGGGCTGATAGTCTTATCTTACAGGTCATCTGCGGGTGGCCTGAATAGGTACGATTTACTAACTGGAAGAGGCACTAAATGAACACGATTAACATCGCTAAGAACGACTTCTCTGACATCGAACTGGCTGCTATCCCGTTCAACACTCTGGCTGACCATTACGGTGAGCGTTTAGCTCGCGAACAGTTGGCCCTTGAGCATGAGTCTTACGAGATGGGTGAAGCACGCTTCCGCAAGATGTTTGAGCGTCAACTTAAAGCTGGTGAGGTTGCGGATAACGCTGCCGCCAAGCCTCTCATCACTACCCTACTCCCTAAGATGATTGCACGCATCAACGACTGGTTTGAGGAAGTGAAAGCTAAGCGCGGCAAGCGCCCGACAGCCTTCCAGTTCCTGCAAGAAATCAAGCCGGAAGCCGTAGCGTACATCACCATTAAGACCACTCTGGCTTGCCTAACCAGTGCTGACAATACAACCGTTCAGGCTGTAGCAAGCGCAATCGGTCGGGCCATTGAGGACGAGGCTCGCTTCGGTCGTATCCGTGACCTTGAAGCTAAGCACTTCAAGAAAAACG  >  NC_001604.1/3073‑3666`  

                                                                                                                                                                                                                                                                                                          `|`                                                                                                                                                                                                                                                                                                         
   `aCTTGACGCAATGTTAATGGGCTGATAGTCTTATCTTACAGGTCATCTGCGGGTGGCCTGAATAGGTACGATTTACTAACTGGAAGAGGCACTAAATGAACACGATTAACATCGCTAAGAACGACTTCTCTGACATCGAACTGGCTGCTATCCCGTTCAACACTCTGGCTGACCATTACGGTGAGCGTTTAGCTCGCGAACAGTTGGCCCTTGAGCATGAGTCTTACGAGATGGGTGAAGCACGCTTCCGCAAGATGTTTGAGCGTCAACTTAAAGCTGGTGAGGTTGCGGATAACGCTgc                                                                                                                                                                                                                                                                                                    >  1:501003/1‑301 (MQ=255)`  
               `gTTAATGGGCTGATAGTCTTATCTTACATGTCATCTGCGGGTGGCCTTAATAGGTACGATTTACTAACTGGAAGAGTCACTAAATGAACACGATTAACATCGCTAAGAACGACTTCTCTGACATCGAACTGGCTGCTATCCCGTTCAACACTCTGGCTGACCATTACGGTGAGCGTTTAGCTCGCGAACAGTTGGCCCTTGAGCATGAGTCTTACGAGATGGGTGAAGCACGCATCCGCAAGATGTTTGAGCGTCAACTTAAAGCTGGTGAGGTTGCGGATAACGCTGCCGCCAAGCctct                                                                                                                                                                                                                                                                                        >  1:662259/1‑301 (MQ=255)`  
               `gTTAATGGGCTGATAGTCTTATCTTACAGGTCATCTGCGGGTGGCCTGAATAGGTACGATTTACTAACTGGAAGAGGCACTAAATGAACACGATTAACATCGCTAAGAACGACTTCTCTGACATCGAACTGGCTGCTATCACGTTCAACACTCTGGCTGACCATTACGGTGAGCGTTTAGCTCGCGAACAGTTGGCCCTTGAGCATGAGTCTTACGAGATGGGTGAAGCACGCTTCCGCAAGATGTTTGAGCGTCAACTTAAAGCTGGTGAGGTTGCGGATACCGCTGCCGCCAAGCctcc                                                                                                                                                                                                                                                                                        >  1:699874/1‑300 (MQ=255)`  
                   `ataagagacaggTCTTATCTTACAGGTCATCTGCGGGTGGTCTGAATAGGTACGATTTACTAACTGGAAGAGGCACTAAATGAACACGATTAACATCGCTAAGAACGACTTCTCTGACATCGAACTGGCTGCTATCCCGTTCAACACTCTGGCTGACCATTACGGTGAGCGTTTAGCTCGCGAACAGTTGGCCCTTGAGCATGAGTCTTACGAGATGGGTGAAGCACGCTTCCGCAAGATGTTTGAGCGTCAACTTAAAGCTGGTGAGGTTGTGGATAACGCTGCCGCCAAGCCTCtcatc                                                                                                                                                                                                                                                                                    <  1:588767/290‑1 (MQ=255)`  
                   `ataagagacaggTCTTATCTTACAGGTCATCTGCGGGTGGCCTGAATAGGTACGATTTACTAACTGGAAGAGGCACTAAATGAACACGATTAACATCGCTAAGAACGACTTCTCTGACATCGAACTGGCTGCTATCCAGTTCAACACTCTGGCTGACCATTACGGTGAGCGTTTAGCTCGCGAACAGTTGGCCCTTGAGCATGAGTCTTACGAGATGGGTGAAGCACGCTTCCGCAAGATGTTTGAGCGTCAACTTAAAGCTGGTGAGGTTGCGGATAACGCTGCCGCCAAGCCTCtcatc                                                                                                                                                                                                                                                                                    <  1:746751/290‑1 (MQ=255)`  
                   `aTGGGCTGATAGTCTTATCTTACAGGTCATCTGCGGGTGGCCTGAATAGGTACGATTTACTAACTGGAAGAGGCACTAAATGAACACGATTAACATCGCTAAGAACGACTTCTCTGACATCGAACTGGCTGCTATCCCGTTCAACACTCTGGCTGACCATTACGGTGAGCGTTTAGCTCGCGAACAGTTGGCCCTTGAGCATGAGTCTTACGAGATGGGTGAAGCACGCTTCCGCAAGATGTTTGAGCGTCAACTTAAAGCTGGTGAGGTTGCGGATAACGCTGCCACCAAGCCTCtcatc                                                                                                                                                                                                                                                                                    <  1:477794/301‑1 (MQ=255)`  
                     `gggCTGATAGTCTTATCTTACAGGTCATCTGCGGGTGGCCTGAATAGGTACGATTTACTAACTGGAAGAGGCACTAAATGAACACGATTAACATCGCTAAGAACGACTTCTCTGACATCGAACTGGCTGCTATCCCGTTCAACACTCTGGCTGACCATTACGGTGAGCGTTTAGCTCGAGAACAGTTGGCCATTGAGCATGAGTCTTACGAGATGGGTGAAGCACGCTTCCGCAAGATGTTTGAGCGTCAACTTAAAGCTGGTGAGGTTGCGGATAACGCTGCCGCCAAGCCTCTCATAAc                                                                                                                                                                                                                                                                                  <  1:648805/301‑1 (MQ=255)`  
                      `ggCTGATAGTCTTATCTTACAGGTCATCTGCGGGTGGCCTGAATAGGTACGATTTACTAACTGGAAGAGGCACTAAATGATCACGATTAACATCGCTAAGAACGACTTCTCTGACATCGAACTGGCTGCTATCCCGTTCAACACTCTGGCTGACCATTACGGTGAGCGTTTAGCTCGCGAACAGTTGGCCCTTGAGCATGAGTCTTACGAGATGGGTGAAGCACGCTTCCGCAAGATGTTTGAGCGTCAACTTAAAGCTGGTGAGGTTGCGGATAACGCTGCCGCCAAGCCTCTCATCACt                                                                                                                                                                                                                                                                                 >  1:694155/1‑301 (MQ=255)`  
                        `cTGATAGTCTTATCTTACAGGTCATCTGCGGGTGGCCTGAATAGGTACGATTTACTAACTGGAAGAGGCACTAAATGAACACGATTAACATCGCTAAGAACGACTTCTCTGACATCGAACTGGCTGCTATCCCGTTCAACACTCTGGCTGACCATTACGGTGAGCGTTTAGCTCGCGAACAGTTGGCCCTTGAGCATGAGTCTTACGAGATGGGTGAAGCACGCTTCCGCAAGATGTTTGAGCGTCAACTTAAAGCTGGTGAGGTTGCGGATAACGCTGCCGCCAAGCCTATCATCACTAc                                                                                                                                                                                                                                                                               >  1:74264/1‑301 (MQ=255)`  
                        `agatgtgtataagagacagggTCATCTGCGGGTGGCCTGAATAGGTACGATTTACTAACTGGAAGAGGCACTAAATGAACACGATTAACATCGCTAAGAACGACTTCTCTGACATCGAACTGGCTGCTATCCCGTTCAACACTCTGGCTGACCATTACGGTGAGCGTTTAGCTCGCGAACAGTTGGCCCTTGAGCATGAGTCTTACGAGATGGGTGAAGCACGCTTCCGCAAGATGTTTGAGCGTCAACTTAAAGCTGGTGAGGTTGCGGATAACGCTGCCGCCAAGCCTCTCATCACTAc                                                                                                                                                                                                                                                                               <  1:617256/282‑1 (MQ=255)`  
                         `tGATAGTCTTATCTTACAGGTCATCTGTGGGTGGCCTGAATAGGTACGATTTACTAACTGGAAGAGGCACTAAATGAACACGATTAACATCGCTAAGAACGACTTCTCTGACATCGAACTGGCTGCTATCCCG‑TCAACACTCTGGCTGACCATTACGGTGAGCGTTTAGCTCGCGAACAGTTGGCCCTTGAGCATGAGTCTTACGAGATGGGTGAAGCACGCTTCTGCAAGATGTTTGAGCATCAACTTAAAGCTGGTGAGGTTGCGGATAACGCTGCCGCCAAGCCTCTCATCACTAccc                                                                                                                                                                                                                                                                             <  1:133972/301‑1 (MQ=255)`  
                          `gagatgtgtataagagacagcATCTGCGGGTGGCCTGAATAGGTACGATTTACTAACTGGAAGAGGCACTAAATGAACACGATTAACATCGCTAAGAACGACTTCTCTGACATCGAACTGGCTGCTATCCCGTTCAACACTCTGGCTGACCATTACGGTGAGCGTTTAGCTCGCGAACAGTTGGCCCTTGAGCATGAGTCTTACGAGATGGGTGAAGCACGCTTCCGCAAGATGTTTTAGCGTCAACTTAAAGCTGGTGAGGTTGCGGATAACGCTGCCGCCAAGCCTCTCATCACTAccc                                                                                                                                                                                                                                                                             <  1:166008/281‑1 (MQ=255)`  
                          `gATAGTCTTATCTTACAGGTCATCTGCGGGTGGCCTGAATAGGTACGATTTACTAACTGGAAGAGGCACTAAATGAACACGATTAACATCGCTAAGAACGACTTCTCTGACATCGAACTGGCTGCTATCCCGTTCAACACTCTGGCTGACCATTACGGTGAGCGTTTAGCTCGCGAACAGTTGGCCCTTGAGCATGAGTCTTACGAGATTGGTGAAGCACGCTTCCGCAAGATGTTTGAGCGTCAACTTAAAGCTGGTGAGGTTGCGGATAACGCTGCCGCCAAGCCTCTCATCACTAccc                                                                                                                                                                                                                                                                             <  1:177986/301‑1 (MQ=255)`  
                          `ctcgtagatggggataagagacagtGCGGGTGGCCTGAATAGGTACGATTAACTAACTTGAAGAGGCACTAAATGAACACGTTTAACATCGCTAAGAACGACTTCTCTGACATCGAACTGGCTGCTATCCCGTGCAACACTCTGGCTGACCATTACGGTGAGCGTTTAGCTCGCAAACAGTTGGCCCTTGAGCATGAGTCTTACGAGATGGGTGAAGCACGCTTCCGCAAGATGTTTGAGCGTCAACTTAAAGCTGGTGAGGTTGCGGATAACGCTGCCGCCAAGCCTCTCATCACTAccc                                                                                                                                                                                                                                                                             <  1:303462/277‑1 (MQ=255)`  
                          `agagacagtatcttaCAGGTCATCTGCGGGTGGCCTGAATAGGTACGATTTACTAACTGGAAGAGGCACTAAATGAACACGATTAACATCGCTAAGAACGACTTCTCTGACATCGAACTGGCTGCTATCCCGTTCAACACTCTGGCTGACCATTACGGTGAGCGTTTAGCTCGCGAACAGTTGGACATTGAGCATGAGTCTTACGAGATGGGTGAAGCACGCTTCCGCAAGATGTTTGAGCGTCAACTTAAAGCTGGTGAGGTTGCGGATAACGCTGCCGCCAAGCCTCTCATCACTAccc                                                                                                                                                                                                                                                                             <  1:687935/293‑1 (MQ=255)`  
                               `tcttatcttaCAGGTCATCTTCGGGTGGCCTGAATAGGTACGATTTACTAGCTGGAAGAGGCACTAAATGAACACGATTAACATCGCTAAGAACGACTGCTCTGACATCGAACTGGCTGCTATCCCGTTCAACACTCTGGCTGACCATTACGGTGAGCGTTTAGCTCGCGAACAGTTGGCCCTTGAGCATGAGTCTTACGAGATGGGTGAAGCACGCTTCCGCAAGATGTTTGAGCATCAACTTAAAGCTGGTGAGGTTGCGGATAACGCTGCCGCCAAGCCTCTAATCACTACCCTACTc                                                                                                                                                                                                                                                                        <  1:811281/301‑1 (MQ=255)`  
                               `tcttatcttaCAGGTCATCTGCGGGTGGCCTGAATAGGTACGATTTACTAACTGGAAGAGGCACTAAATGAACACGATTAACATCGCTAAGAACGACTTCTCTGACATCGAACTGGCTGCTATCCCGTTCAACACTCTGGCTGACCATTACGGTGAGCGTTTAGCTCGCGAACAGTTGGCCCTTGAGCATGAGTCTTACGAGATGGGTGAAGCACGCTTCCGCAAGATGTTTGAGCGTCAACTTAAAGCTGGTGAGGTTGCGGATAATGCTGCCGCCAAGCCTCTCATCACTACCCTACTc                                                                                                                                                                                                                                                                        <  1:566303/301‑1 (MQ=255)`  
                               `tcttatcttaCAGGTCATCTGCGGGTGGCCTGAATAGGTACGATTTACTAACTGGAAGAGGCACTAAATGAACACGATTAACATCGCTAAGAACGACTTCTCTGACATCGAACTGGCTGCTATCCCGTTCAACACTCTGGCTGACCATTACGGTGAGCGTTTAGCTCGCGAACAGTTGGCACTTGAGCATGAGTCTTACGAGATGGGTGAAGCACGATTCCGCAAGATGTTTGAGCGTCAACTTAAAGCTGGTGAGGTTGCGGATAACGCTGCCGCCAAGCCTCTCATCACTACACTACTc                                                                                                                                                                                                                                                                        <  1:116425/301‑1 (MQ=255)`  
                                 `ctcggagatgtgtataagggacagggCCTGAATAGGTACGATTTACTAACTGGAAGAGGCACTAAATGAACACGATTAACATCGCTAAGAACGACTTCTCTGACATCGAACTGGCTGCTATCCCGTTCAACACTCTGGCTGACCATTACGGTGAGCGTTTAGCTCGCGAACAGTTGGCCCTTGAGCATGAGTCTTACGAGATGGGTGAATCACGCTTCCGCAAGATGTTTGAGCGTCAACTTAAAGCTGGTGAGGTTGCGGATAACGCTGCCGCCAAGCCTCTCATCACTACCCTACTccc                                                                                                                                                                                                                                                                      <  1:185968/277‑1 (MQ=255)`  
                                   `atcttaCAGGTCATCTGCGGGTGGCCCGAATAGGTACGATTTACTAACTGGAAGAGGCACTAAATGAACACGATTAACATCGCTAAGAACGACTTCTCTGACATCGAACTGGCTGCTATCCCGTTCAACACTCTGTCTGACCATTACGGTGAGCGTTTAGCTCGCGAACAGTTGGCCCTTGAGCATGAGTCTTACGAGATGGGTGAAGCACGCTTCCGCAAGATGTTTGAGCGTCAACTTAAAGCTGGTGAGGTTGCGGATAACGCTGCCGCCACGCCTCtcatctgtctcttatacacat                                                                                                                                                                                                                                                                    >  1:50932/1‑285 (MQ=255)`  
                                      `ttaCAGGTCATCTGCGGGTGGCCTGAATAGGTACGATTTACTAACTGGAAGAGGCACTAAATGAACACGATTAACATCGCTAAGAACGACTTCTCTGACATCGAACTGGCTGCTATCCCGTTCAACACTCTGGCTGACCATTACGGTGAGCGTTTAGCTCGCGAACAGTTGGCCCTTGAGCATGAGTCTTACGAGATGGGTGAAGCACGCTTCCGCAAGATGTTTGAGCGTCAACTTAAAGCTGGTGAGGTTGCGGATAACGCTGCCGCCAAGCCTCTCATCACTACCCTACTCCCTAAga                                                                                                                                                                                                                                                                 <  1:310906/301‑1 (MQ=255)`  
                                      `cggagatgtgtataagagacagcTGAATAGGTACGATTTACTAACTGGAAGAGGCACTAAATGAACACGATTAACATCGCTAAGAACGACTTCTCTGACATCGAACTGGCTGCTATCCCGTTCAACACTCTGGCTGACCATTACGGTGAGCGTTTAGCTCGCAAACAGTTGGCCCTTGAGCATGAGTCTTACGAGATGGGTGAAGCACGCTTCCGCAAGATGTTTGAGCGTCAACTTAAAGCTGGTGAGGTTGCGGATAATGCTGCCGCCAAGCCTCTCATCACTACCCTACTCCCTAAga                                                                                                                                                                                                                                                                 <  1:81639/279‑1 (MQ=255)`  
                                       `taCAGGTCATCTTCGGGTGGCCTGAATAGGTACGATTTACTAACTGGAAGAGGCACTAAATGAACACGATTAACATCGCTAAGAACGACTTCTCTGACATCGAACTGGATGCTATCCCGTTCAACACTCTGGCTGACCATTACGGTGAGCGTTTAGCTCGCGAACAGTTGGCCCTTGAGCATGAGTCTTACGAGATGGGTGAAGCACGCTTCCGCAAGATGTTTGAGCGTCAACTTAAAGCTGGTGAGGTTGCGGATAACGCTGCCGCCAAGCCTCTCATCACTACCCTACTCCCTAAgat                                                                                                                                                                                                                                                                <  1:776302/301‑1 (MQ=255)`  
                                             `tCATCTGCGGGTGGCCTGATTAGGTACGATTTACTAACTGGAAGAGGCACTAAATGAACACGATTAACATCGCTAAGAACGACTTCTCTGACATCGAACTGGCTGCTATCCCGTTCAACACTCTGGCTGACCATTACGGTGAGCGTTTAGCTAGCGAACAGTTGGCCCTTGAGCATGAGTCTTACGAGATGGGTGAAGCACGCTTCCGAAAGATGTTTGAGCGTCAACTTAAAGCTGGTGATGTTGCGGATAACGCTGCCGCCAAGCCTCTCATCACTACCCTACTCCCTAAGATGATTGc                                                                                                                                                                                                                                                          <  1:280650/301‑1 (MQ=255)`  
                                               `aTCTGTGGGTGGCCTTAATAGGTACGATTTACTAACTGGAAGAGGCACTAAATGAACACGATTAACATCGCTAAGAACGACTTCTCTGACATCGAACTGGCTGCTATCCCGTTCAACACTCTGGCTGACCATTACGGTGAGCGTTTAGCTCGCGAACAGTTGGCCCTTGAGCATGAGTCTTACGAGATGTGTGAAGCACGCTTCCGCAAGATGTTTGAGCGTCAACTTAAAGCTGGTGAGGTTGCGGATAACGCTGCCGCCAAGCCTCTCATCACTACCCTACTACATAAgatgctgtctc                                                                                                                                                                                                                                                        >  1:432269/1‑294 (MQ=255)`  
                                                `tCTGCGGGTGGCCTGAATAGGTACGATTTACTAACTGGAAGAGGCACTAAATGAACACGATTAACATCGCTAAGAACGACTTCTCTGACATCGAACTGGCTGCTATCCCGTTCAACACTCTGGCTGACCATTACGGTGAGCGTTTAGCTCTCGAACAGTTGGCCCTTGAGCATGAGTCTTACGAGATGGGTGAAGCACTCTTCCGCAAGATGTTTGAGCGTCAACTTAAAGCTGGTGAGGTTGCGGATAACGCTGCCGCCAAGCCTCTCATCACTACCCTACTCCCTAAGATGATTGCACg                                                                                                                                                                                                                                                       >  1:254795/1‑301 (MQ=255)`  
                                                      `ggTGGCCTGAATAGGTACGATTTACTAACTGGAAGAGGCACTAAATGAACACGATTAACATCGCTAAGAACGACTTCTCTGACATCGAACTTGCTGCTATCCCGTTCAACACTCTGGCTGACCATTACGGTGAGCGTTTAGCTCGTGAACAGTTGGCCCTTGAGCATTAGTCTTACGAGATGGGTGAAGCACGCTTCCGCAAGATGTTTGAGCGTCAACTTAAAGCTGGTGAGGTTGCGGATAACGCTGCCGCCAAGCCTCTCATCACTACCCTACTcctgtctcttatacacatctccga                                                                                                                                                                                                                                                 >  1:147595/1‑279 (MQ=255)`  
                                                         `ggCCTGAATAGGTACGATTTACTAACTGGAAGAGGCACTAAATGAACACGATTAACATCGCTAATAACGACTTCTCTGACATCGAACTGGCTGCTATCCCGTTCAACACTCTGGCTGACCATTACGGTGAGCGTTTAGCTCGCGAACAGTTGGCCCTTGAGCATGAGTCTTACGAGATGGGTGAAGCACGCTTCCGCAAGATGTTTGAGCGTCAACTTAAAGCTGGTGAGGTTGCGGATAATGCTTCCGCCAAGCCTCTCATCACTACCCTACTCCCTAAGATGATTGCACGCATCAACgc                                                                                                                                                                                                                                              >  1:578035/1‑300 (MQ=255)`  
                                                         `ggCCTGAATAGGTACGATTTACTAACTGGAAGAGGCACTAAATGAACACGATTAACATCGCTAAGAACGACTTCTCTGACATCGAACTGGCTGCTATCCCGTTCAACACTCTGGCTGACCATTACGGTGAGCGTTTAGCTCGCGAACAGTTGGCCCTTGAGCATGAGTCTTACGAGATGGGTGAAGCACGCTTCCGCAAGACGTTTGAGCGTCAACTTAAAGCTGGTGAGGTTGCAGATAACGCTGCCGCAAAGCCTCTCATCACTACCATACTCCCTAAGATGATTGCACGCATCAACGa                                                                                                                                                                                                                                              >  1:781397/1‑301 (MQ=255)`  
                                                          `gCCTGAATAGGTACGATTTACTAACTGGAAGAGGCACTAAATGAACACGATTAACATCGCTAAGAACGACTTCTCTGACATCGAACTGGCTGCTATCCCGTTCAACACTCTGGCTGACCATTACGGTGAGCGTTTAGCTCGCGAACAGTTGGTCCTTGAGCATGCGTATTACGAGATGGGTGAAGCACGCTTCCGCCAGATGTTTGAGCGTCAACTTAAAGCTGGTGAGGTTGCGGATAACGCTGCCGCCAAGCCTCTCATCACTACCCTACTCCCTAAGATGCTTGCACGCATCAACGaa                                                                                                                                                                                                                                             >  1:135763/1‑300 (MQ=255)`  
                                                              `gAATAGGTACGATTTACTAACTGGAAGAGGCACTAAATGAACACGATTAACATCGCTAAGAACGACTTCTCTGACATCGAACTGGCTGCTATCCCGTTCAACACTCTGGCTGACCATTACGGTGAGCGTTTAGCTCGCGAACAGTTGGCCCTTGAGCATGAGTCTTACGAGATGGGTGAAGCACGCTTCCGCAAGATGTTTGAGCGTCAACTTAAAGCTGGTGAGGTTGCGGATAACGCTGCCGCCAAGCCTCTCATCACTACCCTACTACCTAAGATGATTGCACGCATCAACGACTggg                                                                                                                                                                                                                                         >  1:492801/1‑300 (MQ=255)`  
                                                                   `tgtataagagacagaCTGGAAGAGGCACTAAATGAACACGATTAACATCGCTAAGAACGACTTCTCTGACATCGAACTGGCTGCTATCCCGTTCAACACTCTGGCTGACCATTACGGTGAGCGTTTAGCTCGCGAACAGTTGGCACTTGAGCATGAGTCTTACGAGATGGGTGAAGCACGCTTCCGCAAGATGTTTGAGCGTCAACTTAAAGCTGGTGAGGTTGCGGATAACGCTGCCGCCAAGCCTCTCATCACTACCCTACTCCCTAAGATGATTGCACGCATCAACGACTGGTTTGAg                                                                                                                                                                                                                                    <  1:684874/287‑1 (MQ=255)`  
                                                                   `ggTATGATTTACTAACTGGAAGAGGCACTAAATGAACACGATTAACATCGCTAAGAACGACTTCTCTGACATCGAACTGGCTGCTTTCCCGTTCAACACTCTGGCTGACCATTACGGTGAGCGTTTAGCTCGCGAACAGTTGGCCCTTGAGCATGAGTCTTACGAGATTGGTGAAGCACGCTTCCGCAAGATGTTTGAGCGTCAACTTAAAGCTGGTGAGGTTGCGGATAACGCTGCCGCCAAGCCTCTCATCACTACCCTACTCCCTAAGATGATTGCACGCATCAACGACTGGTTTGAg                                                                                                                                                                                                                                    >  1:202239/1‑301 (MQ=255)`  
                                                                    `gTACGATTTACTAACTGGAAGAGGCACTAAATGAACACGATTAACATCGCTAAGAACGACTTCTCTGACATCGAACTGGCTGCTATCCCGTTCAACACTCTGGCTGACCATTACGGTGAGCGTTTAGCTCGCGAACAGTTGGCCCTTGAGCATGAGTCTTACGAGATGGGTGAAGCACGCTTCCGCAAGATGTTTGAGCGTAAACTTAAAGCTGGTGAGGTTGCGGATAACGCTGCCGCCAAGCCTCTCATCACTACCCTACTCCCTAAGATGATTGCACGCATCAACGAcctgtctcttt                                                                                                                                                                                                                                   >  1:454420/1‑291 (MQ=255)`  
                                                                    `gTACGATTTACTAACTGGAAGAGGCACTAAATGAACACGATTAACATCGCTAAGAACGACTTCTCTGACATCGAACTGGCTGCTATCCCGTTCAACACTCTGGCTGACCATTACGGTGAGCGTTTAGCTCGCGAACAGTTGGCCATTGAGCATGAGTCTTACGAGAAGGGTGAAGCACGCTTCCGCAAGATGTTTGAGCGTCAACTTAAAGCTGGTGAGGTTGCGGATAATGCTGCCGCCAAGCCTCTCATCACTACCCTACTCCCTAAGATGATTGCACGCATCAACGACTGGTTTGAgg                                                                                                                                                                                                                                   <  1:571049/301‑1 (MQ=255)`  
                                                                     `gtataagagacagcTGGAAGAGGCACTAAATGAACACGATTAACATCGCTAAGAACGACTTCTCTGACATCGAACTTGCTGCTATCCCGTTCAACACTCTGGCTGACCATTACGGTGAGCGTTTAGCTCGCGAACAGTTGGCCCTTGAGCATGAGTCTTACGAGATGGGTGAAGCACGCTTCCGCAAGATGTTTGAGCGTCAACTTAAAGCTGGTGAGGTTGCGGATAACGCTGCCGCCAAGCCTCTCATCACTACCCTACTCCCTAAGATGATTGCACGCATCAACGACTGGTTTGAGGa                                                                                                                                                                                                                                  <  1:229421/288‑1 (MQ=255)`  
                                                                      `aCGATTTACTAACTGGAAGAGGCACTAAATGAACACGATTAACATCGCTAAGAACGACTTCTCTGACATCGAACTGGCTGCTATCCCGTTCAACACTCTGGCTGACCATTACGGTGAGCGTTTAGCTCGCGAACAGTTGGCCCTTGAGCATGAGTCTTACGAGATGGGTGAAGCACGCTTCCGCAAGATGTTTGAGCGTCAACTTAAAGCTGGTGAGGTTGCGGATAACGCTGCCGCCAAGCCTCTCATCACTACCCTACTCACTAAGATGATTGCACGCATCAACGACTGGTTTGAGGac                                                                                                                                                                                                                                 >  1:95320/1‑300 (MQ=255)`  
                                                                              `cTAACTGGAAGAGGCACTAAATGAACACGATTAACATCGCTAAGAACGACTTCTCTGACATCGAACTGGCTGCTATCCCGTTCAACACTCTGGCTGACCATTACGGTGAGCGTTTATCTCGCGAACAGTTGGCCCTTGAGCATGAGTCTTACGAGATGGGTGAAGCACGCTTCCGCAAGATGTTTGAGCGTCAACTTAAAGCTGGTGAGGTTGCGGATAATGCTGCCGCCAAGCCTCTCATCACTACCCTTCTCCCTAAGATGATTGCACGCATCAACGACTGGTTTGAGGAAGTGAAAGc                                                                                                                                                                                                                         <  1:763134/301‑1 (MQ=255)`  
                                                                                  `cTGGAAGAGGCACTAAATGAACACGATTAACATCGCTAAGAACGACTTCTCTGACATCGAACTGGCTGCTATCCCGTTCAACACTCTGGCTGACCATTACGGTGAGCGTTTAGCTCGCGAACAGTTGGCCCTTGAGCATGAGTCTTACGAGATTGGTGAAGTACGCTTCCGCAAGATGTTTGAGCGTCAACTTAAAGCTGGTGAGGTTGCGGATAATGCTGCCGCCAAGCCTCTCATCACTACCCTACTCCCTAAGATGATTGCACGCATCAACGACTGGTTTGAggctgtctcttataca                                                                                                                                                                                                                     >  1:31241/1‑287 (MQ=255)`  
                                                                                    `ggggctcggagatgtgtataagagacagcATCGCTAAGAACGACTTCTCTGACATCGAACTGGCTGCTATCCCGTTCAACACTCTGGCTGACCATTACGGTGAGCGTTTAGCTCGCGAACAGTTGGCCCTTGAGCATGAGTCTTACGAGATGGGTGAATCACGCTTCTGCAAGATGTTTGAGCGTCAACTTAAAGCTGGTGAGGTTGCGGATAATGCTGCCGCCAAGCCTCTCATCACTACCCTACTCCCTAAGATGATTGCACGCATCAACGACTGGTTTGAGGAAGTGAAAGCTAAgcg                                                                                                                                                                                                                   <  1:384220/273‑1 (MQ=255)`  
                                                                                          `ggCACTAAATGAACACGATTAACATCGCTAAGAACGACTTCTCTGACATCGAACTGGCTGCTATCCCGTTCAACACTCTGGCTGACCATTACGGTGAGCGTTTAGCTCGCGAACAGTTGGCCCTCGAGCATGAGTCTTACGAGATGGGTGAAGCACGCTTCCGCAAGATGTTTGAGCGTCAACTTAAAGCTGGTGAGGTTGCGGATAATGCTGCCTCCAAGCCTCTCATCACTACCCTACTCCCTAAGATGATTGCACGCATCAACGACTGGTTTGAGGAAGTGAAAGCTAAgcgctgaaa                                                                                                                                                                                                             >  1:105588/1‑296 (MQ=255)`  
                                                                                            `cACTAAATGAACACGATTAACATCGCTAAGAACGACTTCTCTGACATCGAACTGGCTGCTATCCCGTTCAACACTCTGGCTGACCATTACGGTGAGCGTTTAGCTCGCGAACAGTTGGCCCTTGAGCATGAGTCTTACGAGATGGGTGAAGCACGCTTCCGCAAGATGTTTTAGCGTCAACTTAAAGCTGGTGAGGTTGCGGATAACGCTGCCGCCAAGCCTCTCATCACTACCCTACTCCCTAAGATGATTGCACGCATCAACGACTGGTTTGAGGAAGTGAAAGCTAAGCGCGGCAAgc                                                                                                                                                                                                           >  1:795397/1‑301 (MQ=255)`  
                                                                                                         `cGATTAACATCGCTAAGAACGACTTCTCTGACATCGAACTGGCTGCTATCCCGTTCAACACTCTGGCTGACCATTACGGTGAGCGTTTAGCTCGCGAACAGTTGGCCCTTGAGCATGAGTCTTACGAGATGGGTGAAGCACGCTTCCGCAAGATGTTTGAGCGTCAACTTAAAGCTGGTGAGGTTGCGGATAACGCTGCCGCCAAGCCTCTCATCACTACCCTACTCCCTAAGATGATTGCACGCATCAACGACTGGTTTGAGGAAGTGAAAGCTAAGCGCGGCAAACGCCCGACAGCCtc                                                                                                                                                                                              >  1:190016/1‑300 (MQ=255)`  
                                                                                                         `cGATTAACATCGCTAAGAACGACTTCTCTGACATCGAACTGGCTGCTATCCCGTTCAACACTATGGCTGACCATTACGGTGAGCGTTTAGCTCGCGAACAGTTGGCCCTTGAGCATGAGTCTTACGAGATGGGTGAAGCACGCTTCCGCAAGATGTTTGAGCGTCAACTTAAAGCTGGTGAGGTTGCGGATAACGCTGCCGCCAAGCCTCTCATCACTACACTACTCCCTAAGATGATTGCACGCATCAACGACTGGTTTGAGGAAGTGAAAGCTAAGCGCGGCAAGCGCCCGACAGCCtt                                                                                                                                                                                              >  1:519203/1‑301 (MQ=255)`  
                                                                                                           `ggagatgtgtataagagacagttCTCTGACATCGAACTGGCTGCTATCCCGTTCAACACTCTGGCTGACCATTACGGTGAGCGTTTAGCTCGCGAACAGTTGGCCCTTGAGCATGAGTCTTACGAGATGGGTGAAGCACGCTTCCGCAAGATGTTTGAGCGTCAACTTAAAGCTGGTGAGGTTGCGGAAAACGCTGCCGCCAAGCCTCTCATCACTACCCTACTCCCTAAGATGATTGCACGCATCAACGACTGGTTTGAGGAAGTGAAAGCTAAGCGCGGCAAGCGCCCGACAGCCTTcc                                                                                                                                                                                            <  1:714127/280‑1 (MQ=255)`  
                                                                                                             `tAACATCGCTAAGAACGACTTCTCTGACATCGAACTGGCTGCTATCCCGTTCAACACTCTGGCTGACCATTACGGTGAGCGTTTAGCTCGCGAACAGTTGGCCCTTGAGCATGAGTCTTACGAGATGGGTGAAGCACGCTTCCGCAAGATGTTTGAGCGTCAACTTAAAGCTGGTGAGGTTGCGGATAATGCTGCCGCCAAGCCTCTCATCACTACCCTACTCCCTAAGATGATTGCACGCATCAACGACTGGTTTGAGGAAGTGAAAGCTAAGCGCGGCAAGCGCCCGACAGCCTTCCAg                                                                                                                                                                                          <  1:759846/301‑1 (MQ=255)`  
                                                                                                             `tAACATCGCTAAGAACGACTTCTCTGACATCGAACTGGCTGCTATCCCGTTCAACACTCTGGCTGACCATTACGGTGAGCGTTTAGCTCGCGAACAGTTGGCCCTTGAGCATGAGTCTTACGAGATGGGTGAAGCACGCTTCCGCAAGATGTTTGAGCGTCAACTTAAAGCTGGTGAGGTTGCGGATAACGCTGCCGCCAAGCCTCTCATCACTACCCTACTCCCTAAGATGATTGCACGCATCAACGACTGGTTTGAGGAAGTGAAAGATAAGCGCGGCAAGCGCCCGACAGCCTTCCAg                                                                                                                                                                                          <  1:52874/301‑1 (MQ=255)`  
                                                                                                             `tAACATCGCTAAGAACGACTGCTCTGACATCGAACTGGCTGCTATCCCGTTCAACACTCTGGCTGACCATTACGGTGAGCGTTTAGCTCGCGAACAGTTGGCCCTTGAGCATGAGTCTTACGAGATGGGTGAAGCACGCTTCCGCAAGATGTTTGAGCGTCAACTTAAAGCTGGTGAGGTTGCGGATAACGCTGCCGCCAAGCCTCTCATCACTACCATACTCCATAAGATGATTGCACGCATAAACGACTGGTGTGAGGAAGTGAAAGCTAAGCGAGGCAAGCGCCCGACAGCCTTCCAg                                                                                                                                                                                          <  1:215289/301‑1 (MQ=255)`  
                                                                                                                `cATCGCTAAGAACGACTTCTCTGACATCGAACTGGCTGCTAGCCCGTTCAACACTCTGGCTGACCATTACGGTGAGCGTTTAGCTAGCGAACAGTTGGCCCTTGAGCATGAGTCTTACGAGATGGGTGAAGCACGCTTCCGCAAGATGTTTGAGCGTCAACTTAAAGCTGGTGAGGTTGCGGATAACGCTGCCGCCAAGCCTCTCATCACTACCCTACTCCCTAAGATGATTGCACGCATCAACGACTGGTTTGAGGAAGTGAAAGCTAAGCGCGGAAAGCGCCAGACAGCCTTCCAGTTc                                                                                                                                                                                       <  1:819293/301‑1 (MQ=255)`  
                                                                                                                                `ttCTCTGACATCGAACGGGCTGCTATCCCGTTCAACACTCTGGCTGACCATTACGGTGAGCGTTTAGCTCGCGAACAGTTGGCCCTTGAGCATGAGTCTTACGAGATGGGTGAAGCACGCTTCCGCAAGATGTTTGAGCGTCAACTTAAAGCTGGTGAGGTTGTGGATAATGCTTCCGCCAAGCCTCTCATCACTACCCTACTCCCTAAGATGATTGCACGCATCAACGACTGGTTTGAGGAAGTGAAAGCTAAGCGCGGCAAGCGCCCGACAGCCTTCCAGTTCCTGCAAGAAATCAAGc                                                                                                                                                                       <  1:294850/301‑1 (MQ=255)`  
                                                                                                                                  `ctctGACATCGAACTGGCTGCTATCCCGTTCAACACTCTGGCTGACCATTACGGTGAGCGTTTAGCTCGCGAACAGTTGGCCCTTGAGCATGAGTCTTACGAGATGGGTGAAGCACGCTTCCGCAAGATGTTTGAGCGTCAACTTAAAGCTGGTGAGGTTGCGGATAACGCTGCCGCCAAGCCTCTCATCACTACCCTACTCCCTAAGATGATTGCACGCATCAACGACTGGTTAGAGGAAGTGAAAGCTACGCGCGGCACGCGCCCGACAGCCtctgtctcttatacacatctccgagcc                                                                                                                                                                     >  1:710719/1‑275 (MQ=255)`  
                                                                                                                                      `gACATCGAACTGGCTGCTATCCCGTTCAACACTCTGGCTGACCATTACGGTGAGCGTTTAGCTAGCGAACAGTTGGCCCTTGAGCATGAGTCTTACGAGATGGGTGAAGCACGCTTCCGCAAGATGTTTGAGCGTCAACTTAAAGCTGGTGAGGTTGCGGATAATGCTGCCGCCAAGCCTCTCATCACTACACTACTCCCTAAGATGATTGCACGCATCAACGACTGGTTTGAGGAAGTGAAAGCTAAGCGCGGCAAGCGCCCGACAGCCTTCCAGTTCCTGCAAGAAATCAAGCCGGAAg                                                                                                                                                                 >  1:558533/1‑301 (MQ=255)`  
                                                                                                                                          `tCGAACTGGCTGCTATCCCGTTCAACACTCTGGCTGACCATTACGGTGAGCGTTTAGCTCGCGAACAGTTGTCCCTTGAGCATGAGTCTTACGATATGGGTGAAGCACGCTTCCGCAAGATGTTTGAGCGTCAACTTAAAGCTGGTGAGGTTGCGGATAATGCTGCCGCCAAGCCTCTCATCACTACCCTACTCCCTGAGATGATTGCACGCATCAACGACTGGTTTGAGGAAGTGAAAGCTAAGCGCGGCAAGCGCCCGACAGCCTTCCAGTTCCTGCAAGAAATCAAGCCGGAAGCCGt                                                                                                                                                             <  1:152691/301‑1 (MQ=255)`  
                                                                                                                                              `aCTGGCTGCTATCCCGTTCAACACTCTGGCTGACCATTACGGTGAGCGTTTAGCTCGCGAACAGTTGGCCCTTGAGCATGAGTCTTACGAGATGGGTGAAGCACGCTTCCGCAATATGTTTGAGCGTCAACTTAAAGCTGGTGAGGTTGCGGATAATGCTGCCGCCAAGCCTCTCATCACTACCCTACTCCCTAAGATGATTGCACGCATCAACGACTGGTTTGAGGAAGTGAAAGCTAAGCGCGGCAAGCGCCCGACAGCCTTCCAGTTCCTGCAAGAAATCAAGCCGGAAGCCATAGCg                                                                                                                                                         <  1:112019/301‑1 (MQ=255)`  
                                                                                                                                              `aCTGGCTGCTATCCCGTTCAACACTATGGCTGACCATTACAGTGAGCGTTTAGCTCGCGAACAGTTGGCCCTTGAGCATGAGTCTTACGAGATGGGTGAAGCACGCTTCCGCAAGATGTTTGAGCGTCAACTTAAAGCTGGTGAGGTTGCGGATAATGCTGCCGCCAAGCCTCTCATCACTACCCTACTCCCTAAGATGATTGCACGCATCAACGACTGGTTTGAGGAAGTGAAAGCTAAGCGCGGCAAGCGCCCGACAGCCTTCCAGTTCCTGCAAGAAATCAAGCCGGAAGCCGTAGCg                                                                                                                                                         <  1:144303/301‑1 (MQ=255)`  
                                                                                                                                                         `ctcggagatgtgtataagagacagaTTACGGTGAGCGTTTAGCTCGCGAACAGTTGGCCCTTGAGCATGAGTCTTACGAGATGGGTGAAGCACGCTTCCGCAAGATGTTTGAGCGTCAACTTAAAGCTGGTAAGGTTGCGGATAACGCTGCCGCCAAGCCTCTCATCACTACCCTACTCCCTAAGATGATTGCACGCATCAACGACTGGTTTGAGGAAGTGAAAGCTAAGCGCGGCAAGCGCCCGACAGCCTTCCAGTTCCTGCAAGAAATCAAGCCGGAAGCCGTAGCGTACATCACCAt                                                                                                                                              <  1:657666/277‑1 (MQ=255)`  
                                                                                                                                                             `gTTCAACACTCTGGCTGACCATTACGGTGAGCGTTTAGCTCGCGAACAGTTGGCCCTTGAGCATGAGTCTTACGAGATGGGTGAAGCACGCTTCCGCAAGATGTTTGAGCGTCAACTTAAAGCTGGTGAGGTTGCGGATAATGCTGCCGCCAAGCCTCTCATCACTACCCTACTCCCTAAGATGATTGCACGCATCAACGACTGGTTTGAGGAAGTGAAAGCTAAGCGAGGCAAGCGCCCGACAGCCTTCCAGTTCCTGCAAGAAATCAAGCCGGAAGCCGTAGCGTACATCCCCCTTAAg                                                                                                                                          >  1:505363/1‑301 (MQ=255)`  
                                                                                                                                                              `gagatgtgtataagagacagttACGGTGAGCGTTTAGCTCGCGAACAGTTGGCCCTTGAGCATGAGTCTTACGAGATGGGTGAAGCACGCTTCCGCAAGATGTTTGAGCGTCAACTTAAAGCTGGTGATGTTGCGGATAATGCTGCCGCAAAGCCTATCATCACTACCCTACTCCCTAAGATGATTGCACACATCAACGACTGGTTTGAGGAAGTGAAAGCTAAGCGCGGCAAGCGCCCGACAGCCTTCCAGTTCCTGCAAGAAATCAAGCCGGAAGCCGTAGCGTACATCACCATTAAGa                                                                                                                                         <  1:248293/281‑1 (MQ=255)`  
                                                                                                                                                               `tgtgtataagagacagccATTACGGTGAGCGTTTAGCTCGCGAACAGTTGGCCCTTGAGCATGAGTCTTACGAGATGGGTGAAGAACGCTTCCGCAAGATGTTTGAGCGTCAACTTAAAGCTGGTGAGGTTGCGGATAACGCTGCCGCCAAGCCTCTCATCACTACCCTACTCCATAAGATGATTGCACGCATCAACGACTGGTTTGAGGAAGTGAAAGCTAAGCGCGGCAAGCGCCCGACAGCCTTCCAGTTCCTGCAAGAAATCAAGCCGGAAGCCGTAGCGTACATCACCATTAAGAc                                                                                                                                        <  1:488243/285‑1 (MQ=255)`  
                                                                                                                                                                     `ctctGGCTGACCATTACGGTGAGCGTTTAGCTCGCGAACAGTTGGCCCTTGAGCATGAGTCTTACGAGATGGGTGAAGCACGCTTCCGCAAGATGTTTGAGCGTCAACTTAAAGCTGGTGAGGTTGCGGATAACGCTGCCGCCAAGCCTCTCATCACTACCCTACTCCCTAAGATGATTGCACGCATCAACGACTGGTTTGAGGAAGTGAAAGCTAAGCGCGGCAAGCGCCCGACAGCCTTCCAGTTCCTGCAAGAAATCAAGCCGGAAGCCGTAGCGTACATCACCATTAAGAcctgtct                                                                                                                                  >  1:731026/1‑296 (MQ=255)`  
                                                                                                                                                                     `ctctGGCTGACCATTACGGTGAGCGTTTAGCTCGCGAACAGTTGGCCCTTCAGCATGAGTCTTACGAGATGGGTGAAGCACGCTTCCGCAAGATGTTTGAGCGTCAACTTAAAGCTGGTGAGGTTGCGGATAACGCTGCCGCCAAGCCTCTCATCACTACCCTACTCCCTAAGATGATTGCACGCATCAACGACTGGTTTGAGGAAGTGAAAGCTAAGCGCGGCAAGCGCCCGACAGCCTTCCAGTTCCTGCAAGAAATCAAGCCGGAAGCCGTAGCGTACATCCCCAtctgtctcttata                                                                                                                                  >  1:765764/1‑289 (MQ=255)`  
                                                                                                                                                                        `gggCTGACCATTACGGTGAGCGTTTAGCTCGCGAACAGTTGGCCCTTGAGCATGAGTCTTACGAGATGGGTGAAGCACGCTTCCGCAAGATGTTTGAGCGTCAACTTAAAGCTGGTGAGGTTGCTGATAATGCTGCCGCCAAGCCTCTCATCACTACCCTACTCCCTAAGATGATTGCACGCATCAACGACTGGTTTGAGGAAGTGAAAGCTAAGCGCGGCAAGCGCCCGACAGCCTTCCAGTTCCTGCAAGAAATCAAGCCGGAAGCCGTAGCGTACATCACCATTAAGACCACTCTGGc                                                                                                                               <  1:363792/300‑1 (MQ=255)`  
                                                                                                                                                                         `ggCTGACCATTACGGTGAGCGTTTAGCTCGCGAACAGTTGGCCCTTGAGCATGAGTCTTACGAGATGGGTGAAGCACGCTTCCGCAAGATGTTTGAGCGTCAACTTAAAGCTGGTGAGGTTGCGGATAACGCTGCCGCCAAGCCTCTCATCACTACCGTACTCCATAAGATGATTGCACGCATCAACGACTGGTTTGAGGAAGTGAAAGCTAAGCGCGGCAAGCGCCCGACAGCCTTCCAGTTCCTGCAAGAAATCAAGCCGGAAGCCGTAGCGTACATCACCATTAAGACCACTATGGCt                                                                                                                              <  1:600993/301‑1 (MQ=255)`  
                                                                                                                                                                             `gACCATTACGGTGAGCGTTTAGCTCGCGAACAGTTGGCCCTTGAGCATGAGTCTTACGAGATCGGTGAAGCACGCTTCCGCAAGATGTTTGAGCGTCAACTTAAAGCTGGTGAGGTTGCGGATAATGCTGCCGCCAAGCCTCTCATCACTACCCTACTCCCTAAGATGATTTCACGCATCAACGACTGGTTTGAGGAAGTGAAAGCTAAGCGCGGCAAGCGCCCGACAGCCTTCCCGTTCCTGCAAGAAATCAAGCCTGAAGCCGTCGCGTACATCACCATTAAGAcctgtctcttatacc                                                                                                                          >  1:802118/1‑288 (MQ=255)`  
                                                                                                                                                                              `caatgtgcgggtatcttttAGCTCGCGATCAGTTGGCCCTTGAGCATGAGTCTTACGAGATGGGTGAAGCACGCTTCCGCAAGATGTTTGAGCGTCAACTTAAAGCTGGTGAGGTTGCGGATAACGCTGCCGCCAAGCCTATCATCACTACCCTACTCCCTAAGATGATTGCACGCATCAACGACTGGTTTGAGGAAGTGAAAGCTAAGCGCGGCAAGCGCCCGACAGCCTTCCAGTTCCTGCAAGAAATCAAGCCGGAAGCCGTAGCGTACATCACCATTAAGACCACTCTGGCTTGCCt                                                                                                                         <  1:318048/285‑1 (MQ=255)`  
                                                                                                                                                                              `aCCATTACGGTGAGCGTTTAGCTCGCGAACAGTTGGCCCTTGAGCATGAGTCTTACGAGATGGGTGAAGCACGCTTCCGCAAGATGTTTGAGCGTCAACTTAAAGCTGGTGAGGTTGCTGATAACGCTGCCGCCAAGCCTCTCATCACTACCCTACTCCCTAAGATGATTGCACGCATCAACGACTGGTTTGAGGAAGTGAAAGCTAAGCGCGGCAAGCGCCCGACAGCCTTCCAGTTCCTGCAAGAAATCAAGCCGGAAGCCGTAGCGTACATCACCATTAAGACCACTCTGGCTTGCCt                                                                                                                         <  1:372487/301‑1 (MQ=255)`  
                                                                                                                                                                               `ccATTACGGTGAGCGTTTAGCTCGCGAACAGTTGGCCCTTGAGCATGAGTCTTACGAGATGGGTGAAGCACGCTTCCGCAAGATGTTTGAGCGTCAACTTAAAGCTGGTGAGGTTGCTGATAATGCTGCCGCCAAGCCTCTCATCACTACCCTACTCCCTAATATGATTGCACGCATCAACGACTGGTTTGAGGAATTGAAAGCTAAGCGCGGCAAGCGCCCGACAGCCTTCCAGTTCCTGCAAGAAATCAAGCCGGAAGCCGTAGCGTACATACCCATTAAGACCACTCTGGCTTGCCTa                                                                                                                        >  1:784840/1‑301 (MQ=255)`  
                                                                                                                                                                               `ccATTACGGTGAGCGTTTAGCTCGCGAACAGTTGGCCCTTGAGCATGAGTCTTACGAGATGGGTGAAGCACGCTTCCGCAAGATGTTTGAGCGTCAACTTAAAGCTGGTGAGGTTGCGGATAACGCTGCCGCCAAGCCTCTCATCACTACCCTACTCCCTAAGATGATTGCACGCATCAACGACTGGTTTGAGGAAGTGAAAGCTAAGCGCGGCAAGCGCCCGACAGCCTTCCAGTTCCTGCAAGAAATCAAGCCGGAAGCCGTAGCGTACATCACCATTAAGACCACTCTGGCTTGCCTa                                                                                                                        >  1:368403/1‑301 (MQ=255)`  
                                                                                                                                                                               `ccATTACGGTGAGCGTTTAGCTCGCGAACAGTTGGCCCTTGAGCATGAGTCTTACGAGATGGGTGAAGCACGCTTCCGCAAGATGTTTGAGCGTCAACTTAAAGCTGGTGAGGTTGCGGATAACGCTGCCGCCAAGCCTCTCATCACTACCCTACTCCCTAAGATGATTGCACGCATCAACGACTGGTTTGAGGAAGTGAAAGCTAAGCGCGGCAAGCGCCCGACAGCCTTCCAGTTCCTGCAAGAAATCAAGCCAGAAGCCGTAGCGTACATCACCATTAAGACCACTCTGGCTTGCCTa                                                                                                                        >  1:622838/1‑301 (MQ=255)`  
                                                                                                                                                                                  `ttACGGTGAGCGTTTAGCTCGCGAACAGTTGGACCTTGAGCATGAGTCTTACGAGATGGGTGAAGCACGCTTCCGCAAGATGTTTGAGCGTCAACTTAAAGCTGGTGAGGTTGCGGATAACGCTGCCGCCAAGCCTCTCATCACTACCCTACTCCCTAAGATGATTGCACGCATCAACGACTGGTTTGAGGAAGTGAAAGCTAAGCGCGGCAAGCGCCCGACAGCCTTCCAGTTCCTGCAAGAAATCAAGCCGGAAGCCGTAGCGTACATCACCATTAAGACCACTCTGGCTTGCCTAAcc                                                                                                                     <  1:337012/301‑1 (MQ=255)`  
                                                                                                                                                                                       `gTGAGCGTTTAGCTCGCGAACAGTTGGCCCTTGAGCATGAGTCTTACGAGATGGGTGAAGCACGCTTCCGCAAGATGTTTGAGCGTCAACTTAAAGCTGGTGAGGTTGCGGATAACGCTGCCGCCAAGCCTCTCATCACTACCCTACTCCCTAAGATGATTGCACGCATCAACGACTGGTTTGAGGAAGTGAAAGCTAAGCGCAGCAAGCGCCCAACAGCCTTCCAGTTCCTGCAAGAAATCAAGCCGGAAGCCGTAGCGTACATCACCATTAAGACCACTCTGGATTGCCTAACCAGTGc                                                                                                                >  1:619468/1‑301 (MQ=255)`  
                                                                                                                                                                                            `cGTTTAGCTCGCGAACAGTTGGCCCTTGAGCATGAGTCTTACGAGATGGGTGAAGCACTCTTCCGCAAGATGTTTGTGCGTCAACTTAAAGCTGGTGAGGTTGCGGATAACGCTGCCGCCAAGCCTCTCATCACTACCCTACTCCCTAAGATGATTGCACGCATCAACGACTGGTTTGAGGAAGTGAAAGCTAAGCGCGGCAAGCGCCCGACAGCCTTCCAGTTCCTGCAAGAAATCAAGCCGGAAGCCGTAGCGTACATCACCATTAAGACCACTCTGGCTTGCCTAACCAGTGCTGACa                                                                                                           <  1:159907/301‑1 (MQ=255)`  
                                                                                                                                                                                            `cGTTTAGCTCGCGAACAGTTGGCCCTTGAGCATGAGTCTTACGAGATGGGTGAAGCACGCTTCCGCAAGATGTTTGAGCGTCAACTTAAAGCTGGTGAGGTTGCGGATAACGCTGACGCCAAGCCTCTCATCACTACCCTACTCCCTAAGATGATTGCACGCAGCAACGACTGGTTTGAGGAAGTGAAAGCTAAGCGCGGCAAGCGCCCGACAGCCTTCCAGTTCCTGCAAGAAATCAAGCCGGAAGCCGTAGCGTACATCACCATTAAGACCACTCTGGCTTGCCTAACCAGTGCTGACa                                                                                                           <  1:320437/301‑1 (MQ=255)`  
                                                                                                                                                                                                `tAGCTTGCGAACAGTTGGCCCTTGATCATGAGTCTTATGAGATGGGTGAAGCACGCTTCCGCAAGATGTTTGAGCGTCAACTTAAAGCTGGTGAGGTTGCGGATAACGCTGCCGCCAAGCCTCTCATCACTACCCTACTCCCTAAGATGATTGCACGCATCAACGACTGGTTTGAGGAAGTGAAAGCTAAGCGCGGCAAGCGCCCGACAGCCTTCCAGTTCCTGCAAGAAATCAAGCCGGAAGCCGTAGCGTACATCACCATTAAGACCAatctctgtctcttatacacatctccgagccc                                                                                                       >  1:232612/1‑274 (MQ=255)`  
                                                                                                                                                                                                   `cTCGCGAACAGTTGGCCCTTGAGCATGAGTCTTACGAGATGGGTGAAGCACGCTTCCGCAAGATGTTTGAGCGTCAACTTAAAGCTGGTGAGGTTGCGGATAACGCTGCCGCCAAGCCTCTCATCACTACCCTACTCCCTAAGATGATTGCACGCATCAACGACTGGTTTGAGGAAGTGAAAGCTAAGCGCGGCAAGCGCCCGACAGCCTTCAAGTTCCTGCAAGAAATCAAGCCGGAAGCAGTAGCGTACATCACCATTAAGACCACTATGGCTTGCCTAACCAGTGCTGACAATACAAc                                                                                                    <  1:484011/301‑1 (MQ=255)`  
                                                                                                                                                                                                   `cTCGCGAACAGTGGGCCCTTGAGCAGGAGTCTTACGAGATGGGTGAAGCACGCTTCCGCAAGATGTTGGAGCGTCAACTTAAAGCTGGTGAGGTAGCGGATAACGCTGCCGCCAAGCCTCTCATCACTACCCTACTCCCTAAGATGATTGCACGCATCAACGACTGGTTTGAGGAAGTGAAAGCTAAGTGCGGCAAGCGCCCGACAGCCTTCCAGTTCCTGCAAGAAATCAAGCCGGAAGCCGTAGCGTACATCACCATTAAGACCACTCTGGCTTGCCTAACCAGTGCTGACAATACAAc                                                                                                    <  1:541018/301‑1 (MQ=255)`  
                                                                                                                                                                                                           `cAGTTGGCCCTTGAGCATGAGTCTTACGAGATGGGTGAAGCACGCTTCCGCAAGATGTTTGAGCGTCAACTTAAAGCTGGTGAGGTTGCGGATAATGCTGCCGCCAAGCCTCTCATCACTACCCTACTCCCTAAGATGATTGCACGCATCAACGACTGGTTTGAGGAAGTGAAAGCTAAGCGCGGCAAGCGCCCGACAGCCTTCCAGTTACTGCAAGAAATCAAGCCGGAAGCCGTAGCGTACATCACCATTAAGACCACTCTGGCTTGCCTAACCAGTGCTGACactgtctcttatacac                                                                                            >  1:628651/1‑286 (MQ=255)`  
                                                                                                                                                                                                            `ggagatgtgtataagagacagcTTATGAGATGGGTGAAGCACACTTCCGCAAGATGTTTGAGCATCAACTTAAAGCTGGTGAGGTTGCGGATAACGCTGCCGCCAAGCCTCTCATCACTACCCTACTCCATAAGATGATTGCACGCATCAACGACTGGTTTGAGGAAGTGAAAGCTAAGCGCGGCAAGCGCCCGACAGCCTTCCAGTTCCTGCAAGAAATCAAGCCGGAAGCCGTAGCGTACATCACCATTAAGACCACTCTGGCTTGCCTAACCAGTGCTGACAATACAACCGTTCAGGc                                                                                           <  1:438237/280‑1 (MQ=255)`  
                                                                                                                                                                                                             `gTTGGCCCTTGAGCATGAGTCTTACGAGATGGGTGAAGCACGCTTCCGCAAGATGTTTGAGCGTCAACTTAAAGCTGGTGAGGTTGCGGATAACGCTGCCGCCAAGCCTCTAATCACTACCCTACTCCCTAAGATGATTGCACGCATCAACGACTGGTTTGAGGAAGTGAAAGCTAAGCGCGGCAAGCGCCCGACAGCCTTCCAGTTCCTGCAAGAAATCAAGCCGGAAGCCGTAGCGTACATCACCATTAAGACCACTCTGGCTTGCCTAACCAGTGCTGACAATACAACCGTTCAGGCt                                                                                          <  1:356240/301‑1 (MQ=255)`  
                                                                                                                                                                                                                  `cccTTGAGCATGGGTCTTACGAGAGGGGTGAATCACTCTTCCGCAAGATGTTTGAGCGTCAACTTAAAGCTGGTGAGGTTGCGGATAACGCTGCCGCCAAGCCTCTCATCACTACCCTACTCCCTAAGATGATTGCACGCATCAACGACTGGTTTGATGAAGTGAAAGCTAAGCGCGGCAAGCGCCCGACAGCCTTCCAGTTCCTGCAAGAAATCAAGCCGGAAGCCGTAGCGTACATCACCATTAAGACCACTCTGGCTTGCCTAACCAGTGCTGACAATACAACCGTTCAGGCTGTAGc                                                                                     <  1:350216/301‑1 (MQ=255)`  
                                                                                                                                                                                                                     `ttGAGCATGAGTCTTACGAGATGGGTGAAGCACGCTTCCGCAAGATGTTTGAGCGTCAACTTAAAGCTGGTGAGGTTGCGGATAACGCTGCCGCCAAGCCCCTCATCACTACCCTACTCCCTAAGATGATTGCACGCATCAACGACTGGTTTGAGGAAGTGAAAGCTAAGCGCGGCAAGCGCCCGACAGCCTTACAGTTCCTGCAAGAAATCAAGCCGGAAGCCGTAGCGTACATAACCATTAAGACCACTATGGCTTGCCTAACCAGTGGTGACAATACAACCGGTCAGGCTGTAGCAAg                                                                                  >  1:403692/1‑301 (MQ=255)`  
                                                                                                                                                                                                                       `gAGCATGAGGCTGACGAGATGGGTGAAGCACGCTTCCGCAAGATGTTTGAGCGTCAACTTAAAGCTGGTGAGGTTGCGGACAACGCTGCCGCCAAGCCTCTAATCACTACCCTACTCCCTAAGATGAGTGCACGCATCAACGACTGGTTTGAGGAAGTGAAAGCTAAGCGCGGCAAGCGCCCGACAGCCTTCCAGTTCCTGCAAGAAATCAAGCCGGAAGCCGTAGCGTACATAACCATTAAGACCACTCTGGCTTGCCTAACCAGTGCTGACAATACAACCGTTCAGGCTGTAGCAAgcg                                                                                <  1:587581/301‑1 (MQ=255)`  
                                                                                                                                                                                                                                `tCTTATGAGATGGGTGAAGCACGCTTCCGCAAGATGTTTGAGCGTCAACTTAAAGCTGGTGAGGTTGCGGATAACGCTGCCGCCAAGTCTCTCATCACTACCCTACTCCCTAAGATGATTGCACGCATCAACGACTGGTTTGAGGAAGTGAAAGCTAAGCGCGGCAAGCGCCCGACAGCCTTCCAGTTCCTGCAAGAAATCAAGCCGGAAGCTGTAGCGTACATCACCATTAAGACCACTCTGGCTTGCCTAACCAGTGCTGACAATACAACCGTTCAGGCTGTAGCAAGCGCAAtcggtc                                                                       >  1:460785/1‑301 (MQ=255)`  
                                                                                                                                                                                                                                  `ttACGAGATGGGTGAAGCACGCTTCCGCAAGATGTTTGAGCGTCAACTTAAAGCTGGTGAGGTTGCGGATAATGCTGCCGCCAAGCCTCTCATCACTACCCTACTCACTAAGATGATTGCACGCATCAACGACTGGTTTGAGGAAGTGAAAGCTAAGCGCGGCAAGCGCCCGACAGCCTTCCAGTTCATGCAAGAAATCAAGCCGGAAGCCGTAGCGTACATCACCATTAAGACCACTCTGGCTTGCCTAACCAGTGCTGACAATACAAcctgtctcttatacacatctccgagcccacga                                                                     >  1:729011/1‑271 (MQ=255)`  
                                                                                                                                                                                                                                       `agaTGGGTGAAGCACTCTTCCGCAAGATGTTTGAGCGTCAACTTAAAGCTGGTGAGGTTGCGGATAATGCTGCCGCCAATCCTCTCATCACTACCCTACTCCCTAAGATGATTGCACGCATCAACGACTGGTTTGAGGAAGTGAAAGCTAAGCGCGGCAAGCGCCCGACAGCCTTCCAGTTCCTGCAAGAAATCAAGCCGGAAGCCGTAGCGTACATCACCATTAAGACCACTCTGGCTTGCCTAACCAGTGCTGACAATACAACCGTTCAGGCTGTAGCAAGCGCAATCGGTCGGGCCAt                                                                <  1:125589/301‑1 (MQ=255)`  
                                                                                                                                                                                                                                          `tGGGGGAAGCACGCTTCCGCAAGATGTTTGAGCGTAAACTTAAAGCTGGTGAGGTTGCGGATAACGCTGCCGCCAAGCCTCTCATCACTACCCTACTCCCTAAGATGATTGCACGCATCAACGACTGGTTTGAGGAAGTGAAAGCTAAGCGCGGCAAGCGCCCGACAGCCTTCCAGTTCCTGCAAGAAATCAAGCCGGAAGCCGTAGCGTACATCACCATTAAGACCACTCTGGCTTGCCTAACCAGTGCTGACAATACAACCGTTCAGGCTGTAGCAAGCGCAATCGGTCGGGCCATTGa                                                             <  1:467780/301‑1 (MQ=255)`  
                                                                                                                                                                                                                                                  `agagacagccGCAAGATGTTTGAGCGTCAACTTAAAGCTGGTGAGGTTGCGGATAACGCTGCCGCCAAGCCTCTCATCACTACCCTACTCCCTAAGATGATTGCACGCATCAACGACTGGTTTGAGGAAGTGAAAGCTAAGCGCGGCAAGCGCCCGACAGCCTTCCAGTTCCTGCAAGAAATCAAGCCGGAAGCCGTAGCGTACATCACCATTAAGACCACTCTGGCTTGCCTAACCAGTGCTGACAATACAACCGTTCAGGCTGTAGCAAGCGCAATCGGTCGGGCCATTGAGGACGAgg                                                     <  1:219809/293‑1 (MQ=255)`  
                                                                                                                                                                                                                                                     `cGCTTCCGCAAGATGTTTGAGCGTCAACTTAAAGCTGGTGAGGTTGCGGATAATGCTGCCGCCAAGCCTCTCATCACTACCCTACTCCCTAAGATGATTGCACGCATCAACGACTGGTTTGAGGAAGTGAAAGCTAAGCGCGGCAAGCGCCCGACAGCCTTCCAGTTCCTGCAAGAAATCAAGCCGGAAGCCGTAGCGTACATCACCATTAAGACCACTCTGGCTTGCCTAACCAGTGCTGACAATACAACCGTTCAGGCTGTAGCAAGCGCAATCGGTAGGGCCATTGAGGAcctgtctc                                                  >  1:768879/1‑294 (MQ=255)`  
                                                                                                                                                                                                                                                         `tCCGCAAGATGTTTGAGCGTCAACTTAAAGCTGGTGAGGTTGCGGATAATGCTGCCGCCAAGCCTCTCATCACTACCCTACTCCCTAAGATGATTGCACGCATCAACGACTGGTTTGAGGAAGTGAAAGCTAAGCGCGGCAAGCGCCCGACAGCCTTCCAGTTCCTGCAAGAAATCAAGCCGGAAGCCGTAGCGTACATCACCATTAAGACCACTCTGGCTTGCCTAACCAGTGCTGACAATACAACCGTTCAGGCTGTAGCAAGCGCAATCGGTCGGGCCATTGAGGACGAGGCTCGCtt                                              >  1:241750/1‑301 (MQ=255)`  
                                                                                                                                                                                                                                                          `gcGCAAGATGTTTGAGCGTAAACTTAAAGCTGGTGAGGTTGCGGATAACGATGCCGCCAAGCCTATCATCACTACCCTACTCCCTAAGATGATTGCACGCATCAACGACTGGTTTGAGGAAGTGAAAGCTAAGCGCGGCAAGCGCCCGACAGCCTTCCAGTTCCTGCAAGAAATCAAGCAGGAAGCCGTAGCGTACATCACCATTAAGACCACTATGGCTTGCCTAACCAGTGCTGACAATACAACCGTTCAGGCTGTAGCAAGCGCAATCGGTCGGGACATTGAGGACGAGGCTCGCTTc                                             <  1:550479/300‑1 (MQ=255)`  
                                                                                                                                                                                                                                                                   `ttttGAGCGTCACCTTAAAGCTGGTGAGGTTGCGGATAACGCTGCCGCCAAGCCTCTCATCACTACCCTACTCCCTAAGATGATTGCACGCATCAACGACTGGTTTGAGGAAGTGAAAGCTAAGCGCGGCAAGCGCCCGACAGCCTTCCAGTTCCTGCAAGAAATCAAGCCGGAAGCCGTAGCGTACATCTCCATTAAGACCACTCTGGCTTGCCTAACCAGTGCTGACAATACAACCGTTCAGGCTGTAGCAAGCGCAATCGGTCGGGCCATTGAGGACGAGGCTCGCTTCGGTCGTATc                                    <  1:390767/300‑1 (MQ=255)`  
                                                                                                                                                                                                                                                                   `gTTTGCGCGTCAACTTAAAGCTGGTGAGGTTGCGGATAATGCTGCCGCCAAGCCTCTCATCACTACCCTACCCCCTAAGATGATTGCACGCATCAACGACTGGTTTGAGGAAGTGAAAGCTAAGCGCGGCAAGCGCCCGACAGCCTTCCAGTTCCTGCAAGAAATCAAGCCGGAAGCCGTAGCGTACATCACCATTAAGACCACTCTGGCTTGCCTAACCAGTGCTGACAATACAACCGTTCAGGCTGTAGCAAGCGCAATCGGTCGGGCCATTGAGGACGAGGCTCGCTTCGGTCGTATc                                    <  1:806027/301‑1 (MQ=255)`  
                                                                                                                                                                                                                                                                    `tttGAGCGTCAACTTAAAGCTGGGGAGGTTGCGGATAATGCTGCCGCCAAGCCTCTCATCACTACCCTACTCCCTAAGATGATTGCACGCATCAACGACTGGTTTGAGGAAGTGAAAGCTAAGCGCGGCAAGCGCCCGACAGCCTTCCAGTTCCTGCAAGAAATCAAGCCGGAAGCCGTAGCGTACATCACCATTAAGACCACTCTGGCTTGCCTAACCAGTGCTGACAATACAACCGTTCAGGCTGTAGCAAGCGCAATCGGTCGGGCCATTGAGGACGAGGCTCGCTTCGGTCGTATcc                                   <  1:824021/301‑1 (MQ=255)`  
                                                                                                                                                                                                                                                                     `aaagagacagaCTTAAAGCTGGTGAGGTTGCGGATAATGCTGCCGCCAAGCCTCTCATCACTACCCTACTCCCTAAGATGATTGCACGCATCAACGACTGGTTTGAGGAAGTGAAAGCTAAGCGCGGCAAGCGCCCGACAGCCTTCAAGTTCCTGCAAGAAATCAAGCCGGAAGCCGTAGCGTACATCACCATTAAGACCACTCTGGCTTGCCTAACCAGTGCTGACAATACAACCGTTCAGGCTGTAGCAAGCGCAATCGGTCGGGACATTGAGGACGAGGCTCGCTTCGGTCGTATCCg                                  <  1:790616/291‑1 (MQ=255)`  
                                                                                                                                                                                                                                                                          `gtgtctaagagacagggTGAGGTTGCGAATAATGCTGCCGCCAAGCCTCTCATCACTACCATACTCCCTAAGAAGATTGCACGCCTAAACGACTGGTTTGAGGAAGTGAAAGCTAAGCGCGGCAAGCGCCCGACAGCCTTCCAGTGCCTGCAAGAAATCAAGCCGGAAGCCGTAGCGTACATCACCATTAAGACCACTCTGGCTTGCCTAACCAGTGCTGACAATACAACCGTTCAGGCTGTAGCAAGCGCAATCGGTCGGGCCATTGAGGACGAGGCTCGCTTCGGTCGTATCCGTGAcc                             <  1:275291/286‑1 (MQ=255)`  
                                                                                                                                                                                                                                                                                 `ttAAAGCTGGTGAGGTTGCGGATAACGCTGCTGCCAAGCCTCTCATCACTACCCTACTCCCTAAGATGATTGCACGCATCAACGACTGGTTTGAGGAAGTGAAAGCTAAGCGCGGCAAGCGCCCGACAGCCTTCCAGTTCCTGCAAGAAATCAAGCCTGAAGCCGTAGCGTACATCACCATTAAGACCACTCTGGCTTGCCTAACCAGTGCTGACAATACAACCGTTCAGGCTGTAGCAAGCGCAATCGGTCGGGCCATTGAGGACGAGGCTCGCTTCGGTCGTATCCGTGACCTTGAAGc                      <  1:86200/301‑1 (MQ=255)`  
                                                                                                                                                                                                                                                                                              `ggTTGCGGATAACTCTGCCGCCAAGCCTCTCATCACTACCCTACTCCCTAAGATGATTGCACGCATCAACGACTGGTTTGAGGAAGTGAAAGCTAAGCGCGGCAAGCGCCCGACAGCCTTCCAGTTCCTGCAAGAAATCAAGCCGGAAGCCGTAGCGTACATCACCATTAAGACCACTCTGGCTTGCCTAACCAGTGCTGACAATACAACCGTTCAGGCTGTAGCAAGCGCAATCGGTCGGGCCATTGAGGACGAGGCTCGCTTCGGTCGTATCAGTGACCTTGAAGCTAAGCACTTCAAg         <  1:673223/301‑1 (MQ=255)`  
                                                                                                                                                                                                                                                                                                    `ggATAATGCTGCCGCCAAGCCTCTCATCACTACCCTACTCCCTAAGATGATTGCACGCATCAACGACTGGTTTGTGGAAGTGAAAGCTAAGCGCGGCAAGCGCCCGACAGCCTTCCAGTTCCTGCAAGAAATCAAGCCGGAAGCCGTAGCGTACATCACCATTAAGACCACTCTTGCTTGCCTAACCAGTGCTGACAATACAACCGTTCAGGCTGTAGCAAGCGCAATCGGTCGGGCCATTGATGACGAGGCTCGCTTCGGTCGTATCCGTGActgtctcttatacacatctccgagcacc   >  1:100379/1‑274 (MQ=255)`  
                                                                                                                                                                                                                                                                                                    `ggATAATGCTGCCGCCAAGCCTCTCATCACTACCCTACTCCCTAAGATGATTGCACGCATCAACGACTGGTTTGAGGAAGTGAAAGCTAAGCGCGGCAAGCGCCCGACAGCCTTCCAGTTCCTGCAAGAAATCAAGCCGGAAGCCATAGCGTACATCACCATTAAGACCACTCTGGCTTGCCTAACCAGTGCTGACAATACAACCGTTCAGGCTGTAGCAAGCGCAATCGGTCGGGCCATTGAGGACGAGGCTCGCTTCGGTCGTATCCGTGACCTTGAAGCTAAGCACTTCAAGAAAAAc   >  1:421078/1‑301 (MQ=255)`  
                                                                                                                                                                                                                                                                                                     `gATAATGCTGCCGCCAAGCCTCTCATCACTATCCTACTCCCTAAGATGATTGCACGCATCAACGACTGGTTTGAGGCAGTGAAAGCTAAGCGCGGCAAGCGCCCGACAGCCTTCCAGTTCCTGCAAGAAATCAAGCCGGAAGCCGTAGCGTACATCACCATTAAGACCACTCTGGCTTGCCTAACCAGTGCTGACAATACAACCGTTCAGGCTGTAGCAAGCGCAATCGGTCGGGCCATTGAGGACGAGGCTCGCTTAGGTCGTATCCGTGACCTTGAAGCTAAGAACTTCAAGAAAAACg  >  1:638831/1‑301 (MQ=255)`  

                                                                                                                                                                                                                                                                                                          `|`                                                                                                                                                                                                                                                                                                         
`GCAACTTGACGCAATGTTAATGGGCTGATAGTCTTATCTTACAGGTCATCTGCGGGTGGCCTGAATAGGTACGATTTACTAACTGGAAGAGGCACTAAATGAACACGATTAACATCGCTAAGAACGACTTCTCTGACATCGAACTGGCTGCTATCCCGTTCAACACTCTGGCTGACCATTACGGTGAGCGTTTAGCTCGCGAACAGTTGGCCCTTGAGCATGAGTCTTACGAGATGGGTGAAGCACGCTTCCGCAAGATGTTTGAGCGTCAACTTAAAGCTGGTGAGGTTGCGGATAACGCTGCCGCCAAGCCTCTCATCACTACCCTACTCCCTAAGATGATTGCACGCATCAACGACTGGTTTGAGGAAGTGAAAGCTAAGCGCGGCAAGCGCCCGACAGCCTTCCAGTTCCTGCAAGAAATCAAGCCGGAAGCCGTAGCGTACATCACCATTAAGACCACTCTGGCTTGCCTAACCAGTGCTGACAATACAACCGTTCAGGCTGTAGCAAGCGCAATCGGTCGGGCCATTGAGGACGAGGCTCGCTTCGGTCGTATCCGTGACCTTGAAGCTAAGCACTTCAAGAAAAACG  >  NC_001604.1/3073‑3666`

  

Alignment Legend

Aligned base mismatch/match (shaded by quality score): `ATCG/ATCG` `< 3 ≤` `ATCG/ATCG` `< 18 ≤` `ATCG/ATCG` `< 28 ≤` `ATCG/ATCG` `< 37 ≤` `ATCG/ATCG` `< 38 ≤` `ATCG/ATCG`

Unaligned base: `atcg`    Masked matching base: `atcg`    Alignment gap: `‑`    Deleted base: `‑`
